# Visuomotor Learning Enhanced by Augmenting Instantaneous Trajectory Error Feedback during Reaching

**DOI:** 10.1371/journal.pone.0046466

**Published:** 2013-01-30

**Authors:** James L. Patton, Yejun John Wei, Preeti Bajaj, Robert A. Scheidt

**Affiliations:** 1 Bioengineering, University of Illinois at Chicago, Chicago, Illinois, United States of America; 2 Sensory Motor Performance Program, Rehabilitation Institute of Chicago, Chicago, Illinois, United States of America; 3 Departments of Physical Medicine & Rehabilitation, Northwestern University, Chicago, Illinois, United States of America; 4 Department of Biomedical Engineering, Marquette University, Milwaukee, Wisconsin, United States of America; University of Michigan, United States of America

## Abstract

We studied reach adaptation to a 30° visuomotor rotation to determine whether augmented error feedback can promote faster and more complete motor learning. Four groups of healthy adults reached with their unseen arm to visual targets surrounding a central starting point. A manipulandum tracked hand motion and projected a cursor onto a display immediately above the horizontal plane of movement. For one group, deviations from the ideal movement were amplified with a gain of 2 whereas another group experienced a gain of 3.1. The third group experienced an offset equal to the average error seen in the initial perturbations, while a fourth group served as controls. Learning in the gain 2 and offset groups was nearly twice as fast as controls. Moreover, the offset group averaged more reduction in error. Such error augmentation techniques may be useful for training novel visuomotor transformations as required of robotic teleoperators or in movement rehabilitation of the neurologically impaired.

## Introduction

There are many situations in sports, performing arts, physical rehabilitation following brain injury and remote operation of devices (eg. robotically-assisted surgery) where it is desirable to train or retrain individuals to move their limbs in a specific desired manner. Motor learning is strongly driven to reduce kinematic performance errors [1], [2] and in particular, deviations from a straight-line hand path in horizontal planar reaching [3], [4]. Recent experimental evidence has demonstrated that it is possible to train subjects to produce desired movements of the arm [5], [6] or legs [7] by accentuating trajectory errors using robotic forces. Subjects in those studies were exposed to custom-designed force fields that promoted the learning of specific movements by exploiting short-term adaptive processes [8]. While those perturbations were strictly mechanical, it is well known that motor adaptations are also elicited by visuomotor distortions such as those induced by prisms (see [9] for a review) as well as by rotations, stretches and other distortions of the conventional hand-to-screen mapping [10], [11] [3]. Here we sought to investigate whether two types of visual feedback manipulations might enhance motor learning in healthy adult subjects.

Artificial learning systems (e.g., neural networks) frequently exploit error-driven learning techniques such that learning progresses more quickly when error is larger [12]. We tested whether human motor learning might be enhanced by manipulating sensory feedback so that hand path errors in reaching appear larger than they actually are. We consider two approaches: error amplification via manipulation of the visuomotor *gain* and error biasing via the addition of an *error-offset* equal to the average initial error. Previously we have found that subjects adjust their motor commands to compensate for approximately 32% of the hand trajectory error on their prior movement attempt [8], hence the theoretical limit of magnification is approximately 3.1. It is indeed compelling to consider the potential benefits of using accurate sensors from a machine to detect and then elevate perceived error above physiological noise levels. While error amplification may conceivably lead to larger signal-to-noise ratios in sensory feedback, learning could become unstable if the magnification error causes the subject to over-compensate. Because motor variability, sensor inaccuracies and other uncertainties also influence learning [13], [14], [15], error magnification may be practicably limited to gains considerably less than the theoretical limit. On the other hand, adding an *offset* bias to augment error may be equally or more effective because only the average tendencies of error would be amplified, rejecting spurious mistakes. Furthermore, error-offset presents persistent errors throughout training, even as the learner improves. This technique may sustain motivation throughout practice, and hence increase the total amount of learning. However, each approach has its own potential pitfalls: gain augmentation is vulnerable by potentially causing underdamped or even unstable learning, whereas the offset approach is vulnerable by potentially causing learning beyond the goal.

Here in a preliminary investigation, we evaluated these candidates for error augmentation by evaluating the rate and magnitude of hand path error reduction as subjects made point-to-point reaching movements of the unseen arm while holding a horizontal planar robot. Deviations from the ideal, straight-line trajectory were augmented with either a magnification of 2, a magnification of 3.1, or by an offset angular deviation. We hypothesized that motor learning can be enhanced by error augmentation. Specifically, we hypothesized that error enhancement would be most evident in the case of offset error augmentation. We further hypothesized that the magnification factor predicted by learning models [8] to be at the limits of stable learning (3.1) would be less effective than the more modest gain of 2, due to over-compensation. Our results provide support for the use of two of these error augmentation techniques to facilitate the learning of motor tasks, and identify a practical limit on the magnitude of viable gain augmentation. Portions of the work have been presented in conference proceedings [16].

## Methods

Sixteen neurologically normal adults (22–30 years old) gave informed, written consent to participate in this study in accordance with the Northwestern University Institutional Review Board (IRB), which specifically approved this study and follows the principles expressed in the Declaration of Helsinki. The experiment was carried out on a planar manipulandum robot that has been described in detail elsewhere [17]. A real-time control system managed the experiment, controlled the robot, and stored hand position data at 100 Hz. The robot motors removed the inertial effects of the robot linkage, rendering a nearly impedance-free movement of the handle, and no additional forces were implemented.

Seated subjects grasped the handle of the robot and were instructed to make fast and accurate reaching movements from a common, central starting position, stopping in one of six targets distributed around a circle of radius r = 0.1 m. Motion was cued by blanking the starting point and displaying one of the targets onto an opaque screen placed immediately above the plane of handle motion. This screen occluded direct view of the robot linkage and the subject's entire arm. Return movements to the center point were not analyzed, although all feedback conditions during the return were identical to the immediately preceding outward movement. Three targets (90°, 210° and −30° clockwise from anterior from anterior) were used as a training set while the remaining three targets were unpracticed test targets (directly anterior at 0° and at ±120°). On some trials, a cursor was projected just above the hand throughout the trial. On others, cursor motion was rotated 30° counter-clockwise about the starting point relative to hand motion. The target sets were selected such that movements to the training targets after full compensation for the visuomotor rotation would be approximately the same as movements to the test targets without compensation. After each movement, we provided visual and auditory feedback of peak hand speed to encourage subjects to move at consistent and fast rate. Colored targets and tones indicated when peak movement speed was too fast, too slow, or within the desired range (0.45 m/s±0.05 m/s).

Before experimentation, subjects were allowed to become comfortable moving the handle between targets. The experiment itself was conducted in 3 phases. First, a baseline phase of 165 movements established initial performance to each of the six targets. The latter 120 trials of this phase also intermittently evaluated the subject's response to the 30° counter-clockwise rotation (once every 8 movements, randomly presented, and never two in succession). These initial exposure trials assessed the starting error level for the visuomotor transformation condition that was ultimately learned by subjects. Next, a training phase of 390 movements evaluated the time course and extent of adaptation to a constant 30° counter-clockwise rotation. Here, the subjects were divided evenly and randomly into four treatment groups. The first (the control group) learned the visual rotation by itself while the other three groups learned while experiencing one of three error augmentation schemes. For two of these, deviations from the ideal, straight-line trajectory were magnified by a factor of 2 (the *2 group) or the gain of 3.1 (the *3.1 group), which theoretically should cause a complete error correction in a single trial. The remaining group had their error augmented by a counter-clockwise offset rotation (described below). All groups experienced periodic catch-trials – pseudo-randomly presented once in every eight movements – wherein error augmentation was removed (if present) and progress in adapting to the original 30° rotation was evaluated and compared across groups. Subjects trained only on an evenly-spaced subset of three of the six target directions (90, 210 and −30 clockwise from anterior) but were evaluated pre and post training on all target directions to evaluate their ability to generalize what was learned. Finally, all visual rotations and error augmentation were removed during a washout phase of 165 movements. This phase explored the time course of recovery of each subject's original, unperturbed performance during reaching to the training targets.

**Figure 1:**
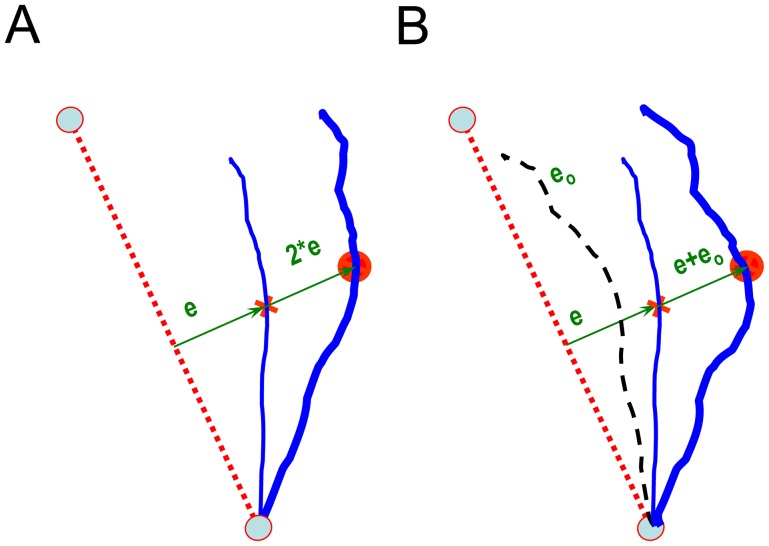
Illustration of the error augmentation strategies. The ideal and actual trajectories are indicated as dotted and thin lines, respectively. At each instant, the cursor (large red dot) is displayed by calculating the current error and either by (**A**) multiplying that error by a gain [in this case a gain of 2] or, (**B**) by adding the offset trajectory *e_0_* to that error. Hence the subject sees the cursor move along the trajectory represented by the thick lines.

For the offset group, the error presented on any given trial was the sum of the instantaneous error ε(r) on that trial and the trial-average error ε_o_(r) generated during initial exposure trials to the same target, where *r* is the hand's radial distance from the starting point (Fig. 1B). The ε_o_(r) trajectories were averaged separately for each of the three movement directions for each subject. In both cases, ε was computed as the perpendicular distance between the ideal straight-line movement and the hand's instantaneous position as a function of *r*. These values were then averaged across trials, within targets. The average errors ε_o_(r) typically began near zero, then grew larger, and then returned near zero at the target. Note that while the *2 and offset conditions may yield similar error feedback signals at the beginning of training, the feedback from these conditions can differ as training progresses. For small errors in hand path, subjects experiencing gain augmentation would see the cursor's movement closely match the desired trajectory, whereas subjects experiencing offset augmentation would continue to perceive substantial errors.

### Data Analysis

**Figure 2:**
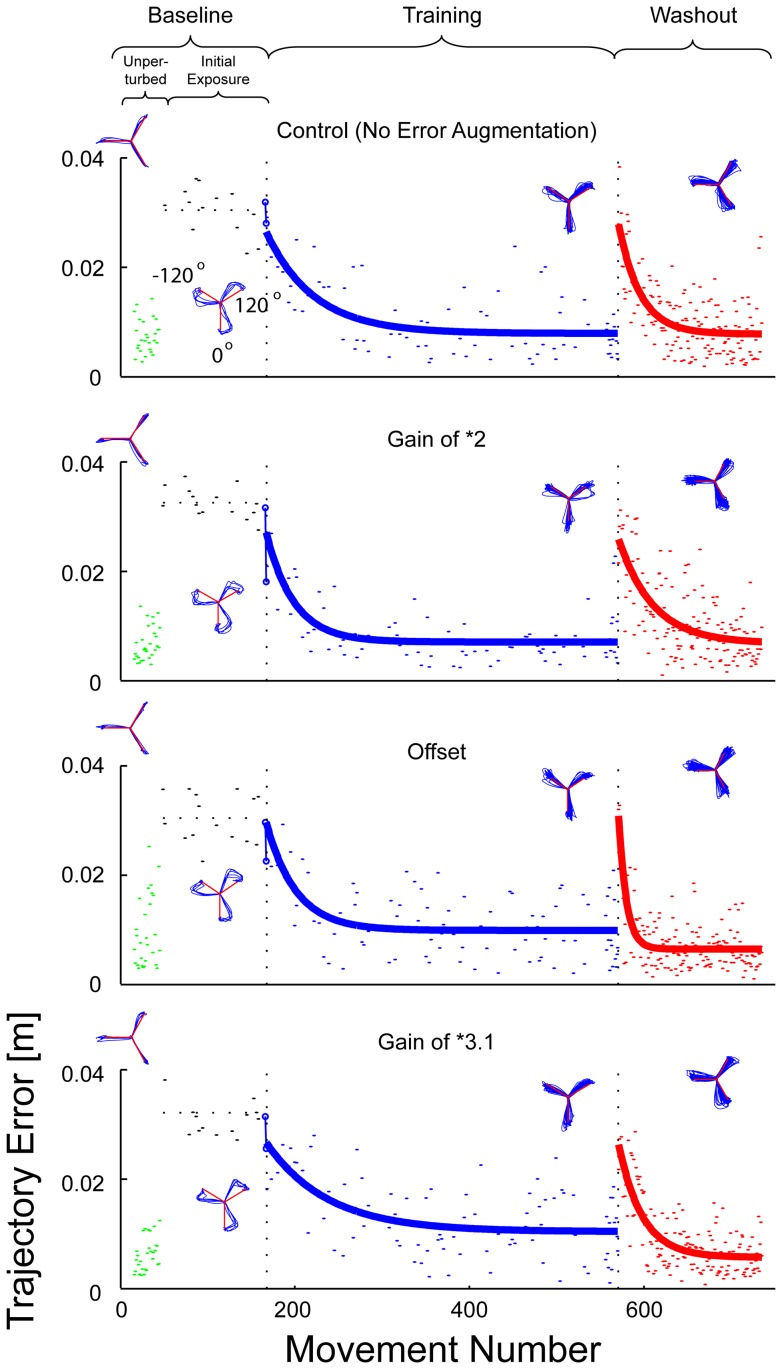
Representative trajectories and learning curves for representative subjects in each group. Each row of plots displays data from a typical subject from each group for the successive phases of the experiment. The insets above each curve show typical movement paths where red lines indicate the path the subjects should have reached to successfully complete the task. Learning and washout phases were each fit to exponential curves (bold blue and red lines). While the training conditions differed for each group, the catch-trials used to fit these curves were performed under identical conditions for all groups (30° rotation with no error augmentation). These random and intermittent catch trials occurred at the same movement number for all groups. Initial error reduction after the first exposure to the distortion is shown as a blue line segment connecting encircled trials.

Baseline conditions for horizontal planar reaching (with no distortion or error augmentation) typically approximate a straight line (Fig. 2, left column; see also [18], [19]). We defined *trajectory error* as the maximum perpendicular distance between the actual hand path and the straight-line path between start and goal positions. Exponential curves were fit to the trial-by-trial error time series using nonlinear Nelder-Mead least-squares regression:
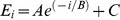
(1)where 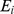 was the trajectory error for the trial 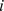 within a training or washout phase, *A* is the amount of learning (the change of the trajectory errors due to training), *B* is the time constant indicating the number of trials for the error to decrease 67% of the way to asymptote, and C is the asymptotic (steady-state) error value.

We planned five statistical tests comparing the quality of learning across the four subject groups. One-way ANOVA compared the *amount* and *rate* of learning (i.e. model parameters A and B respectively from the model fit to individual subject data obtained in the training phase), the amount of steady-state error (model parameter C during training) as well as the amount and rate of washout (i.e. parameters A and B from a separate fit of the model to data obtained after removal of the visuomotor rotation). Post-hoc, Tukey *t*-tests were used to perform pairwise comparisons between groups and were considered statistically significant at the α  = 0.05 level.

## Results

As expected, all subjects in all four groups learned to compensate for the imposed visuomotor rotation. Trajectories were curved on initial exposure to the imposed rotation (Fig. 2 insets) but subjects regained straight-line movements by the end of training. Upon removal of the rotation, trajectories displayed after-effects of learning (i.e. errors in the absence of perturbation) in the direction opposite to those made during the initial exposure phase, thus providing strong evidence that adaptation had indeed taken place. These after-effects washed out over the final washout phase (Fig. 2, right). Initial values of error were not significantly different amongst groups (One-way ANOVA: F_(3,12)_ = 2.55, p = 0.104; see Table 1), and all groups reduced error an average of 68% of the original amount.

**Table 1:** Summary statistics of error values and their changes.

		Initial error	Learning Amount	Steady state	Time constant
Group	N	(m)	(m)	(m)	(movements)
**control**	4	0.029 ± 0.002	0.016 ± 0.002	0.007 ± 0.001	50.9 ± 10.60
*2	4	0.031 ± 0.002	0.014 ± 0.004	0.01 ± 0.002	33.4 ± 7.66
**offset**	4	0.032 ± 0.002	0.022 ± 0.003	0.011 ± 0.002	38.1 ± 4.74
*3.1	4	0.03 ± 0.001	0.016 ± 0.002	0.011 ± 0.001	51.9 ± 14.24

**Figure 3:**
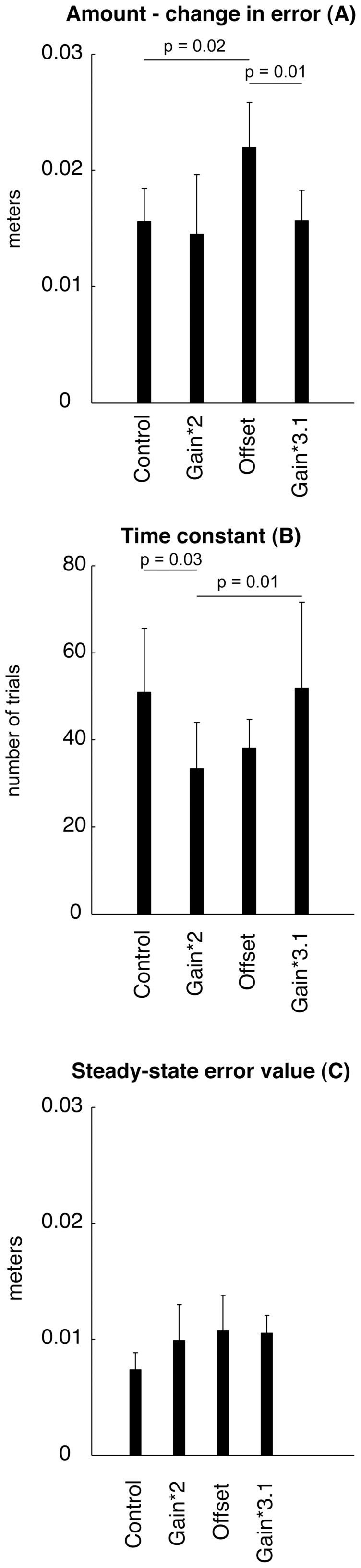
Group results of the curve fitting for all subjects in the four groups according to Eq. 1. The amount of learning (parameter *A*, top), time constant of error decay during learning (parameter B, middle) and steady state value (parameter C, bottom) are shown. Error bars indicate 95% confidence intervals. Horizontal lines indicate significant differences (post-hoc) between groups.

Error augmentation influenced the amount of learning during training (parameter A in Eq 1) (One-way ANOVA: F_(3,12)_ = 4.13, p = 0.032; see Table 1). The Offset group reduced error (22.7±3.6 mm; mean ±1 SD both here and elsewhere) more than the control group (17.5±1.3 mm) whereas error reduction in the other two augmentation conditions (*2: 18.7±3.3 mm; *3.1: 16.9±1.2 mm) did not differ significantly from the controls (Fig. 3A). Error augmentation also influenced the time constant of learning (parameter B in Eq. 1) (One-way ANOVA: F(3,12) = 5.58, p = 0.012; see Table 1). As anticipated, the *2 group proved to learn faster (29.1±10.3 trials) than both control subjects (44.8±8.9 trials) and *3 group subjects (52.1±10.3 trials). The asymptotic level of performance error (parameter C in Equation 1) did not vary across training groups (One-way ANOVA: F(3,12) = 2.12, p = 0.150; see Table 1). One-way ANOVA also found no group-dependent differences in the amount (F_(3,12)_ = 1.90, p = 0.183) or rate (F_(3,12)_ = 1.57, p = 0.247) of washout, and thus the de-adaptation process was not influenced by type of feedback experienced during training. Finally, we found no group-dependent differences in the summed squares of the residuals of the exponential fit (1-way ANOVA F_(3,12)_ = 2.52; p = 0.11), indicating no detectable group differences in both quality of fit or trial-to-trial variability. Residuals averaged 0.94 millimeters. Although the effect size of training condition on the rate and amount of learning was quite large (amount: Cohen's d  = 1.9; rate: Cohen's d  = 1.6), our sample sizes were small and hence it was not possible to verify normality of the sample distributions. We therefore repeated our analysis using non-parametric Kruskal-Wallis tests and found a pattern of significance identical to that reported above using one-way ANOVA.

Interestingly, the *3.1 group did not show any clear benefit over the control group, despite the observation of an increased learning rate for a gain of 2. Two possibilities may explain this loss in performance enhancement at higher error feedback gains. First, *3.1 group subjects might have overcompensated for the highly magnified errors, a pattern of underdamped and oscillating compensation that might lead to less effective learning. A second possibility is that the nervous system may have adapted learning in order to be more “cautious” as it updated its movement plan. Our data appear to support the second option because each subject learned gradually without obvious oscillations or large “jumps” in the time series of errors. Instead, subjects compensated more gradually from trial to trial (such as in Fig. 2, bottom right). To confirm this, we compared the change in signed error between the first two augmentation trials. We found no difference amongst groups (F_(3,12)_ = 1.12; p = 0.38; shown as blue line segments in Fig. 2). Hence learning rates decreased at the higher gain of 3.1, and this reduction occurred within the first few training trials.

## Discussion

This paper presents a preliminary investigation of whether learning of novel visuomotor transformations may be promoted using error augmentation strategies. The smaller time constants for the *2 and Offset groups demonstrate that error augmentation can increase the rate of learning. Manipulating the gain of visual errors appears to be limited in that a gain of 2 shows markedly better performance than a gain of 3.1. In contrast, the Offset group learned significantly more than the other groups, making this an important new tool for enhancing motor performance during learning. Capitalizing on the ability of computers and displays to perform real-time operations on the feedback may be a valid approach to using technology to enhance the motor learning process.

Manipulating visual feedback in order to promote motor adaptation is certainly not a new idea. Numerous studies have shown how the nervous system can be “tricked” into altering its performance by giving altered sensory feedback (e.g., [3], [14], [20], [21], [22], [23], [24]). Altering visual feedback has already been demonstrated to cause subjects to perceive a higher stiffness than actually felt [24], to increase the output force beyond their original strength limits [20], alter vestibular-ocular reflex gains [21] or notice and correct for sensory neglect secondary to stroke [25]. The present study is unique in that it augmented just the elements of visual feedback pertaining to deviations from the straight line while preserving unaltered visual information regarding motion directed toward the target. Our results demonstrate that there is a clear advantage to *distorted reality* feedback, where judicious manipulations of visual information can lead to practical improvements in the extent and rate of learning.

The offset condition allows subjects to adapt to the visually rotated environment more efficiently than the other methods of augmentation tested. In the gain augmentation condition, errors (and therefore augmentation) decrease to zero as training performance asymptotes at the end of training. In contrast, the error bias added to movements in the Offset Group does not decrease to zero with improvement. The Offset group showed a larger average amount of learning (Figure 3A) that was significantly larger than the 3.1 and the Control groups. This finding suggests that sustaining error artificially large can assist in enhancing continued learning when conventional error-based learning becomes smaller and smaller as each attempt diminishes error. Like a back propagation learning algorithm with momentum [12], where changes in performance can continue to occur even when performance errors are zero, offset error augmentation can potentially drive learning beyond 0 error, thus leading subjects to “overlearn” beyond the desired goal. This outcome may be beneficial in situations where subjects do not fully learn, as was the case for the control group, and may also provide a means to achieve more complete learning. However, we note that such over-learning did not appear to occur in the current experiment. While all groups' final steady state errors were not significantly different across groups, the treatment group averages trended slightly larger than controls (Figure 3C) and so a failure to detect significance could be due to a limited sample size. Nonzero steady state errors are a strict measure of “incomplete learning” (Figure 3C, confidence intervals do not encompass zero). In any case, we speculate that a ‘scheduled’ mixture of offset and gain, in which the offset factor is extinguished when the subject learns beyond the goal, may optimize both the rate and extent of motor learning. This question of what ‘schedule’ of offset and gain to use is related to the topics of gain scheduling and adaptive learning rates that are used in neural network and machine learning [26].

Change in errors immediately following exposure to error augmentation was the same for all groups, even though the *3.1 group should theoretically have learned much more than control subjects. Subjects from *3.1 group tended to slow learning rather than over-compensate. This suggests that the nervous system may react to excessively large error signals by decreasing (within about 5 trials) the impact of visual performance errors on motor command updating. Large errors thus may be regarded as outliers by a nonlinear loss function that governs motor adaptation [14]. The finding that catch-trial performance after initial exposure to error augmentation does not differ across groups indicates that the brain does not disregard large errors as non-meaningful or non-deterministic. This finding is in contrast to studies of visual error *reduction* (a manipulation which appears to stifle learning; [23] and studies of visual feedback suppression (which slows the (dis)adaptation process; [27]). These and other studies that induce conflict between sensory modalities suggest that rather than overcompensating and oscillating its errors as it learns, the nervous system can quickly “adapt its adaptation” by re-weighing the interpretation of sensory information if it no longer is perceived reliable [13], [28].

A limitation of the present study is that it did not parametrically examine the effectiveness of gain augmentation over the full range of multiplicative factors. The gain *3.1 in the experiment did no better than the control (gain *1) and worse than gain *2, possibly because the larger gain may have decreased the relative stability of the adaptation process beyond a critical value so that the Gain *3.1 had similar learning to the Control Group. Consequently, an optimal gain must reside between *1 and *3.1. It is possible that the optimal gain may be task-specific and/or dependent on the sensory modality being manipulated to enhance learning. Another limitation is that offset magnitude was not varied in this experiment. Future studies should identify the range of sensory feedback conditions that enhance learning as well as the scheduling of gain and offset strategies that optimize motor learning in specific tasks.

Offset augmentation belongs to the class of task- and subject-specific training techniques suggested to be effective in encouraging neuromotor rehabilitation in cases requiring the learning (or re-learning) of the relationship between motor intent and action, such as in neurorehabilitation after stroke ([29]. These results support and expand on recent work demonstrating that error-augmenting forces were more beneficial than error-reducing forces in restoring reaching performance after stroke [6]. While not all kinds of augmented feedback have proven to be therapeutically beneficial post-stroke [30], it is important that future efforts should explore other potential gain and offset settings for improving motor training following neuromotor injury. The initial findings presented here in healthy subjects suggest that such training approaches might be effective in facilitating motor learning in sports, performing arts, remote device operation, rehabilitation, or in any training situation requiring repetitive practice.
